# Primary Spontaneous Pneumothorax Admitted in Emergency Unit: Does First Episode Differ from Recurrence? A Cross-Sectional Study

**DOI:** 10.1155/2017/2729548

**Published:** 2017-03-30

**Authors:** S. Kepka, J. C. Dalphin, A. L. Parmentier, J. B. Pretalli, M. Gantelet, N. Bernard, F. Mauny, T. Desmettre

**Affiliations:** ^1^Emergency Department, CHRU of Strasbourg, 1 place de l'Hopital, 67091 Strasbourg, France; ^2^UMR 6249 Chronoenvironnement/University of Franche Comté, La Bouloie-UFR Sciences et Techniques, 16 route de Gray, 25030 Besançon Cedex, France; ^3^Department of Respiratory Diseases, CHRU of Besançon, 1 boulevard Fleming, 25030 Besançon, France; ^4^Clinical Methodology Center, CHRU of Besançon, 2 place Saint Jacques, 25030 Besançon, France; ^5^Emergency Department, CHRU of Besançon, 1 boulevard Fleming, 25030 Besançon, France

## Abstract

*Introduction*. Management of primary spontaneous pneumothorax (PSP) consists of immediate resolution of pleural air, or observation, and prevention of recurrence. The risk factors for recurrence remain debated.* Objectives*. We aimed to describe and compare the characteristics of patients presenting a first episode of PSP to those of patients presenting a recurrent PSP, in order to identify factors potentially related to recurrence.* Methods*. We conducted a cross-sectional study including all admissions for PSP in the EDs of fourteen French public hospitals from 2009 to 2013. PSP were classified as a first episode if the patient had no previous history of pneumothorax and as recurrence if a previous episode of spontaneous pneumothorax was documented in the patient's medical records or if a recurrence was identified during the inclusion period. To identify factors potentially associated with recurrence of PSP, multilevel logistic models were fitted.* Results*. During the study period, 918 (61,6%) first episodes and 573 (38,4%) episodes of recurrent PSP were identified. Clinical presentation, age, gender, smoking habits, and use of cannabis were similar in both groups. No clinical factor associated with recurrence was identified by multivariate analysis.* Conclusion*. In this large multicenter study, no clinical factor associated with recurrence was highlighted.

## 1. Introduction

Primary spontaneous pneumothorax (PSP) is generally a benign pathology occurring in young people, and the main risk associated with PSP is recurrence. However, its socioeconomic impact is considered as an important concern. Management consists of immediate resolution of pleural air, or observation, and prevention of recurrence.

Tobacco smoking is the most important risk factor for the occurrence of PSP [1–3]. Furthermore, cannabis smoking is clearly associated with the apparition of bullae and an increased risk of PSP [4, 5]. Recently, in a large epidemiological study, Bobbio et al. found that PSP was more frequent in men and that age of onset appeared to be significantly higher in women than in men [6]. The recurrence rate of PSP varies according to studies, but it is generally accepted to be about 30% [7–9]. Risk factors for a first occurrence are sometimes considered to be risk factors for recurrence. According to the European Respiratory Society's Scientific Committee, smoking might be the only reversible risk factor for recurrence after a first episode of PSP [3]. On the other hand, studies investigating the impact of tobacco and cannabis smoking, gender, or age, on the recurrence of PSP have shown contradictory results [9–11]. However, these studies included small sample sizes [9, 12, 13] and recurrence was often studied at one year as a criteria evaluating treatment success. Data concerning recurrence are therefore incomplete, and the characteristics of patients presenting with recurrent SP are poorly documented.

Therefore, the aim of this study was to describe and compare the characteristics of patients presenting a first episode of PSP to those of patients presenting a recurrent PSP in order to identify factors potentially related to recurrence in a large multicenter French study.

## 2. Methods

**Figure 1:**
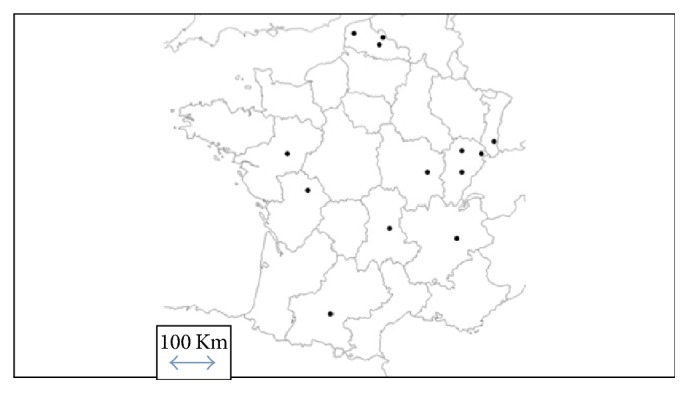
Distribution of participating hospitals in metropolitan France. List of the participating hospitals and local investigators: University Hospital of Angers, France, Mazet B. University Hospital of Besançon, France, Desmettre T. University Hospital of Clermont Ferrand, France, Schmidt J. University Hospital of Dijon, France, Honnard D. University Hospital of Grenoble, France, Carpentier F. University Hospital of Poitiers, France, Lardeur JY. University Hospital of Toulouse, France, Lauque D. Hospital of Belfort, France, Braun JB. Hospital of Béthune, France, Dubard AE. Hospital of Boulogne/Mer, France, Duncan G. Hospital of Lomme, France, Bronet N. Hospital of Mulhouse, France, Goulesque B. Hospital of Roubaix, France, Depelchin A. Hospital of Vesoul, France, El Cadi T.

A multicenter cross-sectional study was performed in the emergency departments (EDs) of fourteen French public hospitals. Data were collected by research assistants in the EDs of seven French university hospitals and seven general (nonacademic) hospitals in 7 of the 13 regions of metropolitan France (Figure 1). Participating departments were spread across the whole of France, covering areas with varying geographic characteristics (such as distance from the coast, or altitude) and different climates.

### 2.1. Study Population

Patients were identified by searching the ED admissions databases of the participating centers. All admissions in EDs between June 1, 2009, and May 31, 2013, with a primary diagnosis of pneumothorax (International Classification of Diseases (ICD) 10 code = J93) were considered. Among the patients thus identified, inclusion criteria were age over 18 years and main diagnosis of PSP confirmed by the physician in charge of patients (ICDcode J93.11). Noninclusion criteria were secondary spontaneous, traumatic pneumothorax and patients older than 50 years (could be considered as secondary pneumothorax). A secondary pneumothorax was defined as presence of any one or more of the following preexisting lung diseases: Chronic Obstructive Pulmonary Disease (COPD), emphysema, or chronic respiratory failure. Traumatic pneumothorax was defined as a history of pneumothorax with thoracic trauma. Among eligible patients, patients with no previous history of pneumothorax were classed as the first episode group (first PSP group). Recurrence was defined as the onset of a new episode of pneumothorax before or during the study period, including ipsilateral or contralateral recurrence. Patients with a previous episode of PSP or with recurrence during the study period were classified in the recurrence group (recurrence PSP group). The information concerning previous episodes of pneumothorax was collected from the medical files of the EDs.

### 2.2. Data Collection and Endpoint

For all patients in both groups, the following sociodemographic variables were collected: age, gender, smoking habits (current smokers, ever smokers or nonsmokers), self-declared use of cannabis, and history of pneumothorax. The circumstances of occurrence of the pneumothorax recorded were period (week/weekend, season), conditions (at rest or during activity), place (at home or not), and time (day from 8 am to 6 pm or night from 6 pm to 8 am). In addition, clinical data, namely, respiratory rate, blood pressure, heart rate, and SpO_2_ in ambient air, were also collected. Numerical pain score at admission was recorded (on a numerical rating scale from 0 to 10). Presence of dyspnea was also investigated by checking the patient's medical file for documented dyspnea. Dyspnea was defined as presence of breathlessness at rest or during exercise.

The objective of the study was to describe and compare the characteristics of patients presenting a first episode of PSP to those of patients presenting a recurrent PSP, in order to identify factors potentially related to recurrence.

### 2.3. Statistical Analysis

Age is presented in categories (<30, 30–39, 40–49, and >50 years). Categorial variables are described as number (percentage). All eligible admissions for PSP were considered for analysis. Some of these admissions were related to the same patients. To take into account the correlated configuration of the data, a three-level hierarchical structure was considered for analysis: admission (*ijk*), patient (*ij*), and hospital (*k*). To identify factors potentially associated with recurrence, multilevel logistic models were fitted. [14]. Multivariate analysis included variables which were significant at a *p* value < 0.20 by univariate analysis, as well as factors known or suspected to be risk factors for SP in the literature (namely, age, gender, smoking, and use of cannabis). The significance threshold was set at 0.05. Analyses were performed using R Version R 3.2.0 (R Foundation for Statistical Computing, Vienna, Austria) and using MlwiN V2.23 software (Centre for Multilevel Modelling, University of Bristol, UK) for multilevel analysis.

### 2.4. Ethical and Legal Considerations

The approval of the local Ethics Committee was obtained on February 26, 2013, under the numbers CCTIRS 13564 and CPP 13-06. The study was approved by the French National authority for the protection of privacy and personal data on December 23, 2014, under the number 913594.

## 3. Results

### 3.1. Study Population

Among the 3086 pneumothoraxes identified, 1491 PSP (48%) were retained for the analysis and 1595 were not eligible (1098 traumatic pneumothorax, 358 secondary pneumothorax, and 139 patients older than 50 years). Among the 1491 PSP, 918 (61.6%) were classified in the first PSP group and 573 (38.4%) in the recurrence group.

### 3.2. Characteristics of FPSP and RPSP Groups

The two groups were similar in terms of age, gender, smoking status, or use of cannabis (Table 1). Conditions of occurrence, such as season and onset at rest or during activity, were similar in both groups. Only place of onset (*p* = 0.004) and time of onset (*p* = 0.041) differ significantly between groups.

Respiratory rate, heart rate, mean blood pressure, SpO_2_, dyspnea, and pain score did not significantly differ between groups (Table 2).

Multivariate analysis adjusted on age, gender, smoking status, and use of cannabis did not identify any variable that was significantly associated with recurrence. When introducing place and time of onset in the model, only place of onset remained significant (OR = 1.4361, 95% CI 1.05–1.97, *p* = 0.025).

## 4. Discussion

To the best of our knowledge, our study is the largest multicenter series to describe characteristics of patients with recurrence and to compare with first episode in 1491 PSP. The main result is that the usual clinical risk factors considered to be linked to occurrence of a first episode of PSP were not found to be associated with recurrence of PSP in this population. The clinical presentation, sociodemographic data, and medical history were similar in both the first PSP and recurrence groups. Only the place of onset was identified in multivariate analysis as a factor associated with recurrence, with pneumothorax more frequently occurring at home in the case of recurrence as compared to first episodes. However, this result must be interpreted with caution in view of the number of missing data (missing data *n* = 423).

Risk factors for the occurrence of PSP are often considered as risk factors for recurrence [3, 9]. Tobacco smoking is the most important risk factor for occurrence of PSP, with a relative risk that is ninefold higher in women and 22-fold in men for smokers compared with nonsmokers [2]. Smoking is thus often presented as a risk factor for recurrence [3, 15]. Even though recurrence seems to occur earlier in smokers [9], we failed to find a significant relation between smoking and recurrence in our study, as in other studies [9, 13]. Furthermore, smokers could have a significant reduced risk of recurrence compared to nonsmokers [13]. An association between the use of cannabis and pneumothorax has been suggested in previous studies [1, 16–18], but no studies have explored the link between cannabis use and PSP recurrence. In our study, about 5% of patients with PSP declared that they were cannabis users, but no relation was found between use of cannabis and recurrent PSP. Others factors such as gender or age are considered as risk factors for recurrence of PSP. PSP appears to be more frequent in men, with a male/female sex ratio of 3.3 : 1, and PSP appears to occur at a higher age in women than in men [6]. Sadikot et al. found that recurrence was more frequent in women than in men [9]. However, the sex ratio did not differ between those with a first occurrence and those with recurrence in our study, in line with the findings of Uramato et al., who also failed to find a significant gender difference between recurrent and nonrecurrent patients after surgery for PSP [11]. Concerning age, although some studies have revealed that it could be a risk factor [10, 19], it was not found to be associated with recurrence of PSP in our study, as in previous reports [9].

This study presents some limitations. The retrospective design and the potential for bias are the main limits. Indeed, several variables such as smoking habits and cannabis use were self-declared and thus subject to bias. We cannot exclude that patients may have underreported their usage, particularly in the recurrence group. Furthermore, some potential factors associated with recurrence were not studied. Other authors have found that a low body mass index is a risk factor for recurrence [9, 13, 20]. We were unable to study this factor because it was not available in medical files of EDs. Furthermore, the relation between BMI and recurrence of PSP is already well demonstrated in these studies. A further limitation is the fact that we did not record precise information concerning the extent of the pneumothorax. Previous reports have suggested that recurrence within one year was associated with a smaller initial size of pneumothorax [21]. Evaluating the size of pneumothorax is not simple, since no official consensus classification exists. Comparisons using the British Thoracic Society (BTS), American College of Chest Physicians (ACCP), and Belgian Society of Pulmonology (BSP) classifications show concordant estimations in only 47% of patients [22]. Finally, our study was focused on clinical risk factors, whereas lung morphological aspects, genetic factors, and therapeutic management were not studied.

Despite its retrospective nature and limitations, this study offers the opportunity to analyze a large population of patients with PSP. Previous studies of the risk factors mainly included smaller samples. For example, Sadikot et al. included 153 patients with 83 recurrences [9]. More recently, Ouanes-Besbes et al. studied the impact of clinical presentation, treatment, and pulmonary scan findings on recurrence of PSP in 80 patients with 15 recurrences [12], while Olesen et al. included 234 patients [13]. While our study does not provide the same information as a cohort study, the statistical power is nonetheless important. We also conducted a multilevel analysis to take into consideration readmissions during the study period, thus accounting for the correlated configuration of the data. Furthermore, the hospitals involved in our study were spread over a large geographical area, in regions with different climatic conditions. However, this study remains the only one to date to include such a wide diversity of regions, climates, and hospitals. Thus, our recruitment stems from a range of different types of hospitals, with local management, and almost exhaustive inclusions. In light of these considerations, it is unlikely that selection bias could explain our results.

One way for physicians to modify the rate of recurrence seems to be the modalities of treatment. Surgery is clearly recommended for recurrence of PSP [23, 24] but it could be debated for a first episode. Video-assisted thoracoscopic surgery (VATS) represents the surgical treatment of choice [3]. Some authors propose immediate VATS after a first episode of PSP in patient who do not heal after simple aspiration [25, 26], because it could reduce the rate of recurrence. A CT-based lung dystrophy severity score has recently been proposed to predict recurrence and could be useful for clinicians to determine patients who could benefit from early surgery after a first episode [27].

In this cross-sectional study, admittances with first episode had similar characteristics to those with recurrence, and no clinical factor associated with recurrence was identified. The place of onset differed between groups. Only a large prospective follow-up of patients admitted for a first episode of PSP would provide a significantly higher standard of proof concerning risk factors of recurrence.

## Figures and Tables

**Table 1:** Patients characteristics and conditions of occurrence in first PSP and recurrence admissions.

Variables	Category	First PSP *N* (%)	Recurrence PSP *N* (%)	*p* value
Gender				0.87
	Female	199 (21.75%)	125 (21.97%)	
	Male	716 (78.25%)	444 (78.03%)	
Age				0.34
	<30 years	623 (68.76%)	369 (65.54%)	
	30–39 years	215 (23.73%)	152 (27.00%)	
	40–49 years	68 (7.51%)	42 (7.46%)	
Smokers				0.30
	Yes	534 (76.83%)	270 (74.38%)	
	No	161 (23.17%)	93 (25.62%)	
Cannabis				0.13
	Yes	54 (5.95%)	22 (3.85%)	
	No	854 (94.05%)	550 (96.15%)	
Date of occurrence				0.46
	Week	704 (76.94%)	427 (74.91%)	
	Weekend	211 (23.06%)	143 (25.09%)	
Season of occurrence				0.25
	Spring	216 (23.61%)	157 (27.54%)	
	Summer	219 (23.93%)	114 (20.00%)	
	Autumn	246 (26.89%)	152 (26.67%)	
	Winter	234 (25.57%)	147 (25.79%)	
Conditions of occurrence				0.90
	Rest	496 (77.38%)	281 (77.41%)	
	Activity	145 (22.62%)	82 (22.59%)	
Place of occurrence				0.004^*∗*^
	At home	378 (55.75%)	252 (64.62%)	
	Other	300 (44.25%)	138 (35.38%)	
Time of occurrence				0.04^*∗*^
	Day	388 (65.76%)	202 (59.24%)	
	Night	202 (34.24%)	139 (40.76%)	

PSP, primary spontaneous pneumothorax.

*p* value: degree of statistical significance in multilevel analysis.

Values with *∗* are the significant variable.

**Table 2:** Clinical presentation in first PSP and recurrence admissions.

Variables	Category	First PSP *N* (%)	Recurrence PSP *N* (%)	*p* value
Respiratory rate (per minute)				0.75
	≤25	271 (83.38%)	184 (84.40%)	
	>25	54 (16.62%)	34 (15.60%)	
Heart rate (beats per minute)				0.24
	≤120	785 (96.44%)	513 (97.71%)	
	>120	29 (3.56%)	12 (2.29%)	
Mean blood pressure (mmHg)				0.19
	<70	15 (1.86%)	5 (0.95%)	
	≥70	793 (98.14%)	520 (99.05%)	
SpO_2_ (%)				0.49
	<90	9 (1.20%)	4 (0.80%)	
	≥90	744 (98.80%)	498 (99.20%)	
Dyspnea				0.52
	yes	345 (39.75%)	204 (38.13%)	
	no	523 (60.25%)	331 (61.87%)	
NRS				0,23
	≤4	300 (49.18%)	209 (53.73%)	
	>4	310 (50.82%)	180 (46.27%)	

PSP, primary spontaneous pneumothorax; NRS, Numerical Rating Scale from 0 to 10.

*p* value: degree of statistical significance in multilevel analysis.

## Abbreviations

PSP: Primary spontaneous pneumothorax
EDs: Emergency departments
COPD: Chronic obstructive pulmonary disease
BTS: British Thoracic Society
ACCP: American College of Chest Physicians
BSP: Belgian Society of Pulmonology.
